# National Trends in Disease Activity for COVID-19 Among Children in the US

**DOI:** 10.3389/fped.2021.700656

**Published:** 2021-07-08

**Authors:** Meghan R. Hutch, Molei Liu, Paul Avillach, Consortium for Clinical Characterization of COVID-19 by EHR (4CE), Yuan Luo, Florence T. Bourgeois

**Affiliations:** ^1^Department of Preventive Medicine, Northwestern University, Chicago, IL, United States; ^2^Department of Biostatistics, Harvard T.H. Chan School of Public Health, Boston, MA, United States; ^3^Department of Pediatrics, Harvard Medical School, Boston, MA, United States; ^4^Department of Biomedical Informatics, Harvard Medical School, Boston, MA, United States; ^5^Computational Health Informatics Program (CHIP), Boston Children's Hospital, Boston, MA, United States

**Keywords:** COVID-19, surveillance, public health, hospitalization, pediatric

## Abstract

Ongoing monitoring of COVID-19 disease burden in children will help inform mitigation strategies and guide pediatric vaccination programs. Leveraging a national, comprehensive dataset, we sought to quantify and compare disease burden and trends in hospitalizations for children and adults in the US.

## Introduction

Data on coronavirus disease 2019 (COVID-19) in children and adolescents remain limited, even though 3.6 million pediatric cases have been reported in the US to date and several disease manifestations unique to children have been identified (1, 2). Understanding the epidemiology of pediatric infections and quantifying the disease burden in children is necessary to inform mitigation strategies, support therapeutic development, and guide pediatric vaccination programs (3).

Several vaccines are currently under study in clinical trials in adolescents and children, with one vaccine approved by the FDA in March 2021 for use in children as young as 12 years. In addition to providing direct health benefits, an important public health goal of immunizing children is achieving herd immunity and removing children as vectors of transmission to vulnerable older adults. Analyses of disease activity across the US will help identify regions where additional pediatric vaccination efforts are needed and enable measurement of the direct and indirect benefits of pediatric vaccination strategies.

In July of 2020, the US Department of Health and Human Services began collecting data on hospitalizations related to COVID-19 for both pediatric and adult patients. Leveraging this comprehensive national datasource, we sought to assess the value of these data to quantify and compare disease burden and trends in hospitalizations for children and adults in the US.

## Methods

We evaluated COVID-19 hospitalizations reported to the US Department of Health and Human Services (4). These data are collected under a federal mandate requiring hospitals to provide utilization and capacity data in order to support the public health response to COVID-19 (5). Data cover all hospitals in the 50 States registered with Centers for Medicare and Medicaid Services, and include daily aggregated counts of new adult and pediatric hospitalizations with laboratory-confirmed COVID-19 at the time of admission. We analyzed all data collected since reporting started July 31, 2020 through April 15, 2021. Counts were aggregated weekly from Friday to Thursday.

We used US Census population estimates to calculate weekly hospitalizations per 100,000 children (0–17 years) and adults, nationally and by region (6). Regions were defined according to US Census classification. To quantify and compare the temporality of disease activity between adult and pediatric populations, we conducted univariate change point analyses on national and regional hospitalization rates for children and adults and identified the date with the largest likelihood of representing an upward change in mean hospitalization rates (7). Descriptive and statistical analyses were performed using R version 3.6.0 (R Foundation; https://github.com/meghutch/COVID-19-Hospitalization-Trends). *P*-values were estimated with parametric bootstrapping and deemed significant when adjusted for Bonferroni correction (*P* < 0.005). Significant *p*-values reflect the likelihood of a change point existing within the time interval.

## Results

**Figure 1:**
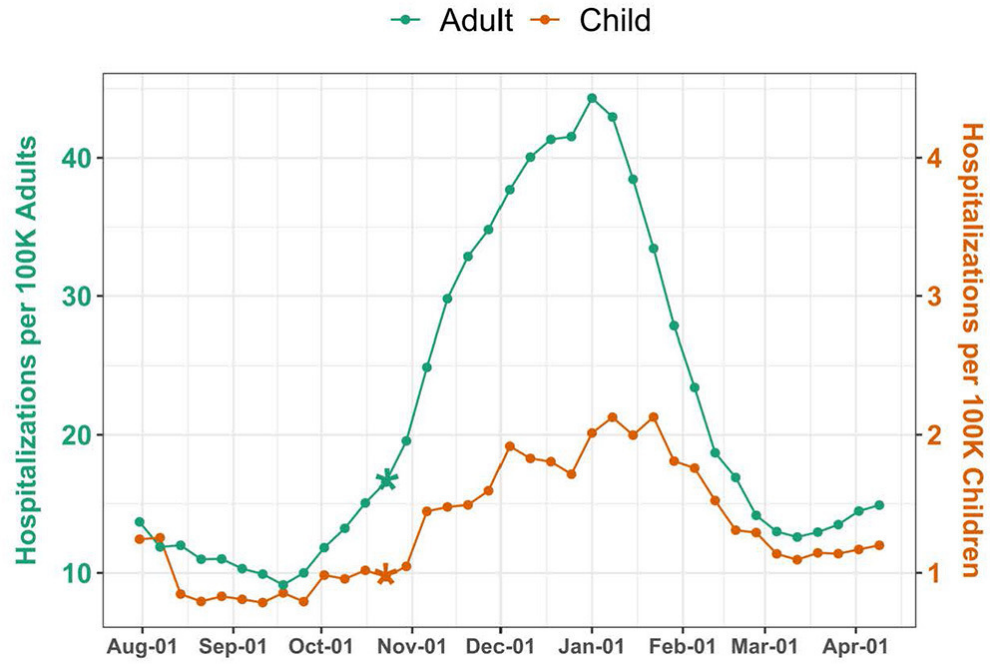
National COVID-19 Hospitalizations among Children and Adults. Hospitalizations were standardized per 100,000 children or adults using US Census population estimates. The scale for pediatric hospitalizations is 10-fold less than for adults. Asterisks indicate significant (*P* < 0.005) change points.

Over the 37-week study period, there were 35,919 pediatric and 2,052,932 adult COVID-19 hospitalizations, corresponding to a weekly median of 906 (IQR 717–1,249) pediatric and 38,675 (IQR 32,338–84,331) adult hospitalizations. National weekly hospitalization rates were 1.2 (IQR 1–1.7) per 100,000 children and 15.1 (IQR 12.6–32.9) per 100,000 adults (Figure 1). Change point analyses identified a significant increase in mean hospitalization rate the week of October 23 for both pediatric and adult populations (*P* = 0.0001 and *P* = 0.0004, respectively). Adult hospitalizations peaked during the week of January 1, at a rate of 44.3 per 100,000 adults, while pediatric hospitalizations peaked the following week, at a rate of 2.1 per 100,000 children.

**Figure 2:**
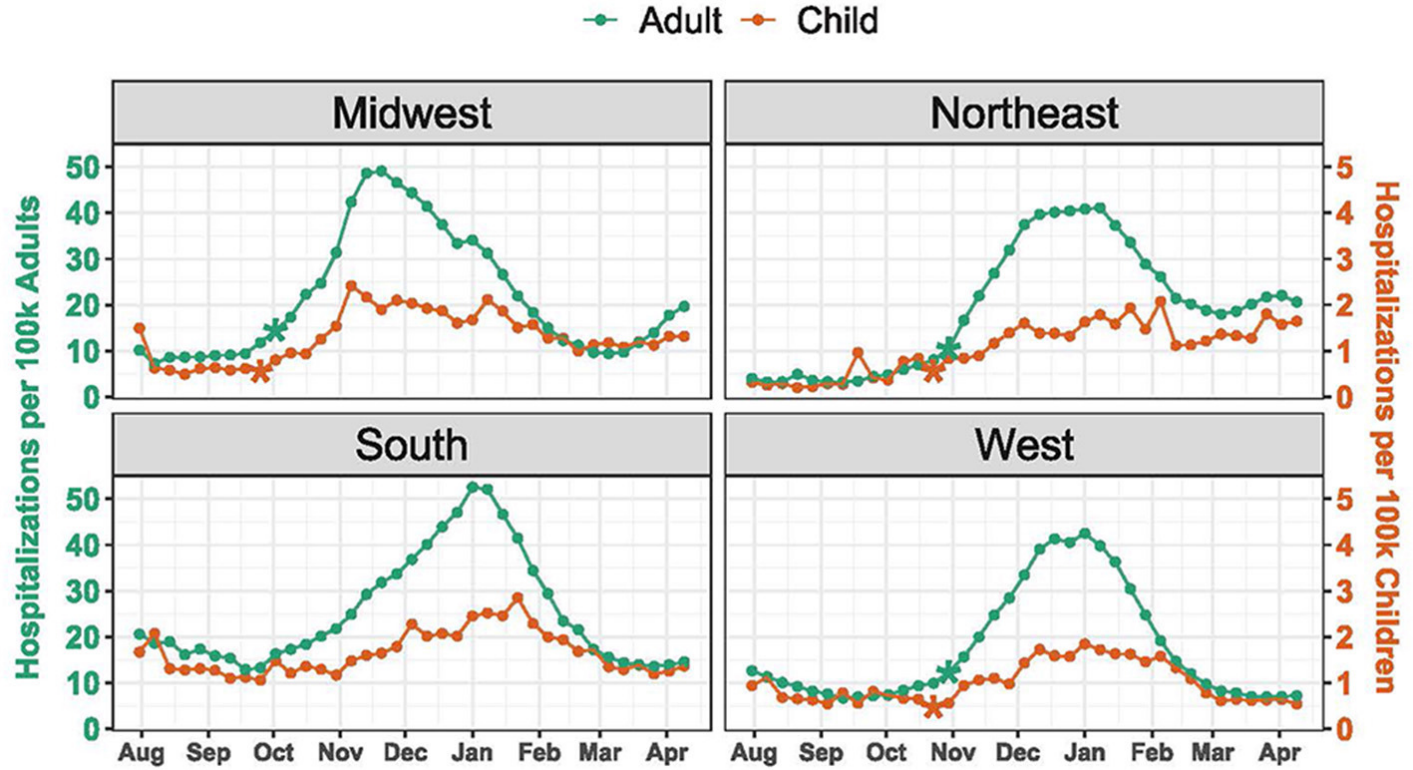
COVID-19 Hospitalization Trends by Region. Hospitalizations were standardized per 100,000 children or adults using US Census population estimates. The scale for pediatric hospitalizations is 10-fold less than for adults. Asterisks indicate significant (*P* < 0.005) change points.

Hospitalization rates and timing of change points varied by region (Figure 2). Median weekly hospitalization rates were highest in the South for both children (1.5 per 100,000 children) and adults (20.2 per 100,000 adults), and lowest for both children and adults in the West (0.9 per 100,000 children and 11.6 per 100,000 adults). The earliest change point for both pediatric and adult hospitalizations occurred in the Midwest the weeks of September 25 (*P* = 0.0002) and October 2 (*P* = 0.0041), respectively. Across the other regions, significant change points were detected during the weeks of October 23 and October 30 for both pediatric and adult hospitalizations. Among children, the earliest peak occurred in the Midwest the week of November 6, while hospitalizations continued to rise in the Northeast until early February.

## Discussion

These national data indicate that peaks in COVID-19 hospitalization rates were 20-fold less for children than adults, though temporal trends were generally similar for pediatric and adult hospitalizations. There was substantial variation across regions, both in rates of hospitalizations observed and timing of changes in disease activity. Our findings highlight the importance of dedicated analyses of pediatric COVID-19 data to measure the impact of the pandemic on children, assess public health measures, and anticipate pediatric healthcare resource needs. Our study builds on prior work by Levin et al. by evaluating a comprehensive data source covering all 50 states and providing a direct comparison of pediatric and adult hospitalization rates through the launch of vaccination programs (8).

Limitations of our study include use of an observational dataset subject to potential misclassification and under-reporting. We also were not able to perform additional patient-level or age group specific analyses as the dataset only identifies patients as pediatric and does not provide detailed demographic or other clinical information.

COVID-19-related hospitalizations are a more robust measure of disease activity than COVID-19 case counts, as they are not subject to biases in testing strategies and represent a relevant health outcome. Evaluation of pediatric and adult hospitalization rates may inform policies for return to work and school activities as adults and children are vaccinated, as well as guide targeted protection of specific population groups. For any policy, the large variations in regional hospitalization rates underscore the need to consider local disease activity in implementation and assessment of mitigation strategies.

## Data Availability Statement

Publicly available datasets were analyzed in this study. These data can be found at: https://healthdata.gov/Hospital/COVID-19-Reported-Patient-Impact-and-Hospital-Capa/g62h-syeh.

## Author Contributions

MH conceptualized and designed the study, collected data, performed the analyses, drafted the initial manuscript, and revised the manuscript. ML performed data analysis and revised the manuscript. PA critically reviewed the manuscript for important intellectual content and revised the manuscript. YL coordinated and supervised data collection, critically reviewed the manuscript for important intellectual content, and revised the manuscript. FB conceptualized and designed the study, coordinated and supervised data collection, critically reviewed the manuscript for important intellectual content, and revised the manuscript. All authors approved the final manuscript as submitted and agree to be accountable for all aspects of the work.

## The Consortium for Clinical Characterization of COVID-19 by EHR (4CE) Members

James R Aaron, Giuseppe Agapito, Adem Albayrak, Mario Alessiani, Danilo F Amendola, Li L. L. J Anthony, Bruce J Aronow, Fatima Ashraf, Andrew Atz, Paul Avillach, James Balshi, Brett K Beaulieu-Jones, Douglas S Bell, Antonio Bellasi, Riccardo Bellazzi, Vincent Benoit, Michele Beraghi, José Luis Bernal Sobrino, Mélodie Bernaux, Romain Bey, Alvar Blanco Martínez, Martin Boeker, Clara-Lea Bonzel, John Booth, Silvano Bosari, Florence T Bourgeois, Robert L Bradford, Gabriel A Brat, Stéphane Bréant, Nicholas W Brown, William A Bryant, Mauro Bucalo, Anita Burgun, Tianxi Cai, Mario Cannataro, Aldo Carmona, Charlotte Caucheteux, Julien Champ, Jin Chen, Krista Chen, Luca Chiovato, Lorenzo Chiudinelli, Kelly Cho, James J Cimino, Tiago K Colicchio, Sylvie Cormont, Sébastien Cossin, Jean B Craig, Juan Luis Cruz Bermúdez, Jaime Cruz Rojo, Arianna Dagliati, Mohamad Daniar, Christel Daniel, Priyam Das, Anahita Davoudi, Batsal Devkota, Julien Dubiel, Loic Esteve, Hossein Estiri, Shirley Fan, Robert W Follett, Paula S. A Gaiolla, Thomas Ganslandt, Noelia García Barrio, Lana X Garmire, Nils Gehlenborg, Alon Geva, Tobias Gradinger, Alexandre Gramfort, Romain Griffier, Nicolas Griffon, Olivier Grisel, Alba Gutiérrez-Sacristán, David A Hanauer, Christian Haverkamp, Bing He, Darren W Henderson, Martin Hilka, Yuk-Lam Ho, John H Holmes, Chuan Hong, Petar Horki, Kenneth M Huling, Meghan R Hutch, Richard W Issitt, Anne Sophie Jannot, Vianney Jouhet, Mark S Keller, Katie Kirchoff, Jeffrey G Klann, Isaac S Kohane, Ian D Krantz, Detlef Kraska, Ashok K Krishnamurthy, Sehi L'Yi, Trang T Le, Judith Leblanc, Andressa RR Leite, Guillaume Lemaitre, Leslie Lenert, Damien Leprovost, Molei Liu, Ne Hooi Will Loh, Sara Lozano-Zahonero, Yuan Luo, Kristine E Lynch, Sadiqa Mahmood, Sarah Maidlow, Adeline Makoudjou, Alberto Malovini, Kenneth D Mandl, Chengsheng Mao, Anupama Maram, Patricia Martel, Aaron J Masino, Maria Mazzitelli, Arthur Mensch, Marianna Milano, Marcos F Minicucci, Bertrand Moal, Jason H Moore, Cinta Moraleda, Jeffrey S Morris, Michele Morris, Karyn L Moshal, Sajad Mousavi, Danielle L Mowery, Douglas A Murad, Shawn N Murphy, Thomas P Naughton, Antoine Neuraz, Kee Yuan Ngiam, Wanjiku FM Njoroge, James B Norman, Jihad Obeid, Marina P Okoshi, Karen L Olson, Gilbert S Omenn, Nina Orlova, Brian D Ostasiewski, Nathan P Palmer, Nicolas Paris, Lav P Patel, Miguel Pedrera Jimenez, Emily R Pfaff, Danielle Pillion, Hans U Prokosch, Robson A Prudente, Víctor Quirós González, Rachel B Ramoni, Maryna Raskin, Siegbert Rieg, Gustavo Roig Domínguez, Pablo Rojo, Carlos Sáez, Elisa Salamanca, Malarkodi J Samayamuthu, L. Nelson Sanchez-Pinto, Arnaud Sandrin, Janaina CC Santos, Maria Savino, Emily R Schriver, Petra Schubert, Juergen Schuettler, Luigia Scudeller, Neil J Sebire, Pablo Serrano Balazote, Patricia Serre, Arnaud Serret-Larmande, Zahra Shakeri, Domenick Silvio, Piotr Sliz, Jiyeon Son, Charles Sonday, Andrew M South, Anastasia Spiridou, Amelia LM Tan, Bryce WQ Tan, Byorn WL Tan, Suzana E Tanni, Deanne M Taylor, Ana I Terriza Torres, Valentina Tibollo, Patric Tippmann, Carlo Torti, Enrico M Trecarichi, Yi-Ju Tseng, Andrew K Vallejos, Gael Varoquaux, Margaret E Vella, Guillaume Verdy, Jill-Jênn Vie, Shyam Visweswaran, Michele Vitacca, Kavishwar B Wagholikar, Lemuel R Waitman, Xuan Wang, Demian Wassermann, Griffin M Weber, Zongqi Xia, Nadir Yehya, William Yuan, Alberto Zambelli, Harrison G Zhang, Daniel Zoeller, Chiara Zucco.

## Conflict of Interest

The authors declare that the research was conducted in the absence of any commercial or financial relationships that could be construed as a potential conflict of interest.

## Abbreviations

COVID-19: Coronavirus disease 2019.
